# Retraction Note: MicroRNA-9 modified bone marrow-derived mesenchymal stem cells (BMSCs) repair severe acute pancreatitis (SAP) via inducing angiogenesis in rats

**DOI:** 10.1186/s13287-023-03419-z

**Published:** 2023-07-28

**Authors:** Daohai Qian, Guodong Song, Zhilong Ma, Guannan Wang, Lei Jin, Minghua Hu, Zhenshun Song, Xiaoming Wang

**Affiliations:** 1 Department of General Surgery, Yijishan Hospital, Wannan Medical College, Wuhu, 241001 Anhui China; 2 Department of General Surgery, Shanghai Tenth People’s Hospital, Tongji University School of Medicine, 301 Yanchang Road, Shanghai, 200072 China; 3 Department of Pharmacology and Pharmaceutical Sciences, USC School of Pharmacy, Los Angeles, CA 90089 USA; 4 Department of Gastroenterology, The Second Affiliated Hospital of Wannan Medical College, Wuhu, 241001 Anhui China

**Retraction Note :Stem Cell Research & Therapy (2018) 9:28**2 10.1186/s13287-018-1022-y

The Editors-in-Chief have retracted this article because of significant concerns regarding a number of Figures presented in this work, which question the integrity of the data. Figure 1 has been found to have been published previously as Figure 1 in the following article [1]. Figure 3A has been found to have been published previously as Figure 4A in the following article [2]. In Figure 2B, there is overlap between the CD31 images of the SAP and the TuD-BMSCs. In Figure 5D, there is overlap between the VECs + miR-9 control and mimic images. Daohai Qian, Lei Jin and Xiaoming Wang do not agree to this retraction. Minghua Hu and Guodong Song agree to this retraction. Zhenshun Song has not explicitly stated whether they agree to this retraction notice. Zhilong Ma, Guannan Wang has not responded to any correspondence from the editor about this retraction.
